# Using Wearable MEG to Study the Neural Control of Human Stepping

**DOI:** 10.3390/s25134160

**Published:** 2025-07-04

**Authors:** Meaghan E. Spedden, George C. O’Neill, Timothy O. West, Tim M. Tierney, Stephanie Mellor, Nicholas A. Alexander, Robert Seymour, Jesper Lundbye-Jensen, Jens Bo Nielsen, Simon F. Farmer, Sven Bestmann, Gareth R. Barnes

**Affiliations:** 1 Department of Imaging Neuroscience, UCL Institute of Neurology, London WC1N 3AR, UK; 2 Department of Neuroscience, Physiology and Pharmacology, University College London, London WC1E 6BT, UK; 3 Department of Bioengineering, Imperial College London, London W12 0BZ, UK; 4 Spinal Cord Injury Center, Balgrist University Hospital, University of Zurich, 8008 Zurich, Switzerland; 5 Translational Neuromodeling Unit, Institute for Biomedical Engineering, University of Zurich & ETH Zurich, 8032 Zurich, Switzerland; 6 Centre for Human Brain Activity (OHBA) University Department of Psychiatry, Oxford OX3 7JX, UK; 7 Department for Clinical and Movement Neuroscience, UCL Queen Square Institute of Neurology, University College London, London WC1N 3BG, UK; s.farmer@ucl.ac.uk; 8 Department of Clinical Neurology, The National Hospital for Neurology and Neurosurgery, Queen Square, London WC1N 3BG, UK; 9 Movement & Neuroscience, Department of Nutrition, Exercise and Sports, University of Copenhagen, 2200 Copenhagen, Denmark; jlundbye@nexs.ku.dk; 10 Department of Neuroscience, University of Copenhagen, 2200 Copenhagen, Denmark; 11 Center for Clinical Neuroscience, Hospital Los Madroños, 28290 Madrid, Spain

**Keywords:** OP-MEG, sensorimotor control, naturalistic neuroimaging

## Abstract

A central challenge in movement neuroscience is developing methods for non-invasive spatiotemporal imaging of brain activity during natural, whole-body movement. We test the utility of a new brain imaging modality, optically pumped magnetoencephalography (OP-MEG), as an instrument to study the spatiotemporal dynamics of human walking. Specifically, we ask whether known physiological signals can be recovered during discrete steps involving large-scale, whole-body translation. Our findings show that by using OP-MEG, we can image the brain during large-scale, natural movements. We provide proof-of-principle evidence for movement-related changes in beta band activity during stepping vs. standing, which are source-localized to the sensorimotor cortex. This work supports the significant potential of the OP-MEG modality for addressing fundamental questions in human gait research relevant to both the physiological and pathological mechanisms of walking.

## 1. Introduction

Capturing high-fidelity brain activity during large-scale, natural movements presents significant methodological challenges. Traditional neuroimaging techniques, such as functional magnetic resonance imaging (fMRI) and cryogenic magnetoencephalography (MEG), require participants to remain still, which limits their ability to capture neural dynamics in natural settings. Electroencephalography (EEG) has been instrumental in advancing research in this field [1,2,3,4,5,6], but its sensitivity to muscle artifacts [7] can pose challenges for movement-related paradigms, and distortions from tissue conductivity can limit its spatial resolution.

Optically pumped magnetometer-based magnetoencephalography (OP-MEG) is a promising alternative for studying brain activity in natural settings [8,9,10,11,12]. Unlike traditional MEG, OPM sensors do not require cryogenic cooling, allowing them to be placed close to the scalp [13] in wearable arrays [14], thereby improving signal strength. The method also benefits from MEG’s advantage of reduced sensitivity to muscle artifacts compared to EEG [7].

However, recording neural activity during movement with OPMs is not without challenges [15,16]. Sensor motion within the ambient magnetic field generates large low-frequency artifacts, which can obscure neural activity or exceed the dynamic range of the sensors. Recent advances in technology and analytical methods [17,18,19,20,21,22] now make it possible mitigate these artifacts significantly. This allows us to shift our perspective from treating movement as a confound [15] to leveraging OPMs for studying the neurophysiology of natural behaviors [23].

One such behavior is walking; a fundamental motor function shared across species that plays a key role in interacting with the environment. While rhythmic walking patterns are partly governed by a spinal network known as the central pattern generator [24,25], human locomotion is more dependent on input from the brain compared to quadrupedal animals [26]. Investigating the neural mechanisms of walking in ecologically valid conditions is critical for understanding both typical and pathological gait, e.g., in individuals with Parkinson’s Disease [27]. By applying OP-MEG to study the cortical control of walking, we can explore how the brain orchestrates locomotion in natural settings.

In this work, we test the utility of this modality to study the spatiotemporal dynamics of human walking using a stepping paradigm. We evaluate whether we can recapitulate neurophysiological signatures of the movement as described with other modalities, aiming to provide physiological proof-of-principle for this methodology as a new instrument available to study large-scale natural movements in humans.

We focus on the movement-related modulation of beta band rhythmicity [28,29], which is known to undergo changes during the planning, initiation, and execution of movement [30]. Our goal is to compare beta band activity during stepping vs. standing and to source localize this activity, leveraging the temporal and spatial resolution offered by OPMs. We hypothesized that beta band activity would decrease during steps compared to standing, with this modulation localized to the sensorimotor cortex, showcasing the capability of OPMs as an imaging modality that can be applied to natural movement.

## 2. Materials and Methods

### 2.1. Participants

We recruited three healthy male participants (Age 55, 33, and 30 years) who provided written, informed consent. The study was approved by the University College London Research Ethics Committee and was conducted in accordance with the Declaration of Helsinki.

### 2.2. Stepping Task

**Figure 1:**
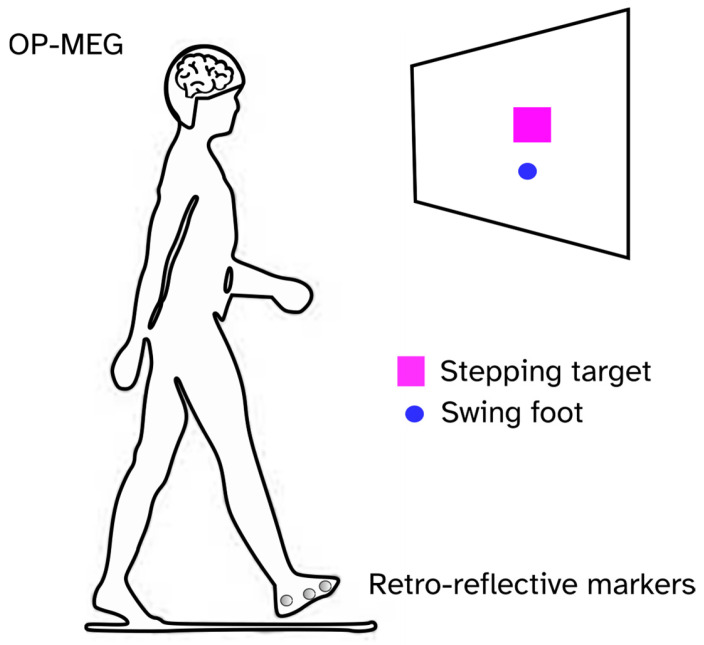
Schematic of the visually guided stepping task. We recorded OP-MEG while participants took single steps forward with the right leg, aiming to hit virtual targets (magenta square). The foot position was tracked using infrared cameras and retro-reflective markers and projected onto the screen as a blue circle. The participants were instructed to adjust their step length to hit the center of the target as precisely as possible.

The participants performed a visually guided stepping task in a magnetically shielded room (MSR) (Figure 1). We have previously used this task to study functional connectivity during stepping [4]. The raw OPM data has been published and is freely available for download [31] and the code is available on Github (https://github.com/meaghanspedden/stepping_opm_data (accessed on 29 June 2025)).

Participants were instructed to take single steps with their right leg, aiming for virtual stepping targets (magenta squares) displayed on a screen in front of them. The position of the stepping foot was tracked using six infrared cameras operating at 120 Hz (Optitrack, Flex 3, Natural Point, Inc., Corvallis, OR, USA), along with retro-reflective markers on the right foot. Foot position data was streamed to MATLAB (version R2023a) via the Motive software (Natural Point, Inc.; NatNet SDK), and visualized on the screen as a blue circle. MATLAB controlled the stimulus presentation and sent a synchronization trigger to the OPM acquisition system when the target was presented. The target distance was randomly selected from three fixed values: the participants’ preferred step length, 5 cm longer than their preferred step length, and 5 cm shorter than their preferred step length. The target position varied only in the anterior–posterior direction. We used three different step lengths to ensure that participants used visual guidance for their steps, and to prevent repetition of the same stepping movement. Before the MEG recording began, participants were given time (approximately 5 min) to practice the task and familiarize themselves with how their foot movements controlled the blue circle on the screen. Participants became proficient in the task quickly, and thus, learning effects during recordings are expected to be minimal.

Each trial lasted approximately 10 s. The trial began when the target appeared on the screen. Participants were instructed to initiate their step with the right foot upon hearing a beep, which served as the go signal. After stepping forward with the right foot, they then brought the left foot forward to place it next to the right foot. The trial ended at that point, and the participant returned to the starting position, which was marked by an open circle on the screen. A total of 5–6 blocks of 30 steps each were recorded, with 5 blocks for participants 1 and 2, and 6 blocks for participant 3. All blocks were recorded on the same day within a single session, and data from each block were concatenated and analyzed together.

### 2.3. Optically Pumped Magnetoencephalography (OP-MEG)

The experiments were conducted in an MSR (Magnetic Shields, Ltd., Staplehurst, UK; internal dimensions: 3 × 4 × 2.2 m), which was degaussed before the start of the experiment. Supplementary Figure S1 shows an example of time and frequency domain empty room data to illustrate the recording environment. The field gradients at the center of our MSR are approximately 1 nT/m and the variation over its cubic meter volume is around 2–3 nT [32]. At standing height (about 1.7 m), we measured the gradient variations over this volume to be approximately 1.2–3.5 nT/m.

**Figure 2:**
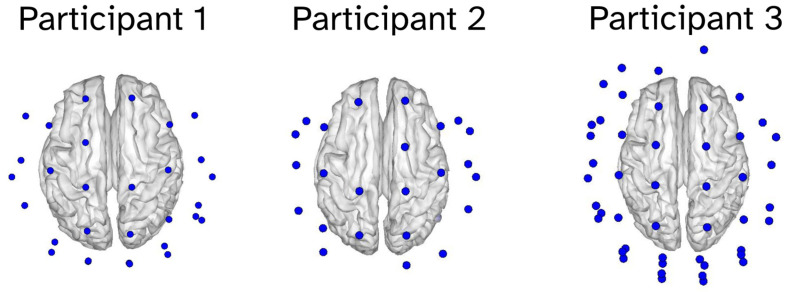
Sensor positions for each participant. The blue circles correspond to OPM sensors. Participant 1 had 30 dual-axis sensors, participant 2 had 27 dual-axis sensors, and participant 3 had 47 triaxial sensors.

Dual-axis and triaxial OP-MEG sensors (QuSpin Inc., Louisville, CO, USA) were mounted in sockets within a custom-built rigid scanner-cast constructed from each participant’s structural MRI. Participant 1 had 30 dual-axis sensors, participant 2 had 27 dual-axis sensors, and participant 3 had 47 triaxial sensors. The number and type of sensor varied based on operational status. Sensor cables were securely attached to the cast. This setup ensures precise co-registration, optimal signal acquisition for any head size, and minimal sensor and cable movement relative to the head. Figure 2 shows the sensor placement for each participant.

For participants 1 and 2, OP-MEG data were recorded using a National Instruments acquisition system with a custom LABVIEW program, operating at a sampling frequency of 6000 Hz. A 500 Hz low-pass FIR filter (60th order, combined with a Kaiser window) was applied before the data were downsampled offline to 2 kHz. The sensors have a dynamic range of ± 4.5 nT. For participant 3, data were acquired using the Neuro-1 system (QuSpin Inc., Louisville, CO, USA), which exclusively used triaxial sensors in an open-loop mode (i.e., no negative feedback applied to the sensors to cancel interference) and operated at a sampling frequency of 1500 Hz. Both acquisition systems had an intrinsic bandwidth of approximately (depending on the vapor temperature and pressure) 0–150 Hz. Additionally, the Neuro-1 system incorporated a high-order digital low-pass FIR filter with a −3 dB cutoff at 150 Hz. Note that the two acquisition systems have been compared, and that results demonstrate both temporal and spatial congruence [18].

### 2.4. Electromyography

EMG from the right tibialis anterior was recorded using two 2 cm electrodes (~2 cm apart; Natus Neurology, Inc., Middleton, WI, USA) and a ground on the right lateral malleolus. In this study, it was used solely to monitor step timing. Cables were routed through waveguides to avoid OPM interference, with signals amplified (×1000), filtered (3–100 Hz, 50 Hz notch), and digitized (1000 Hz) outside the MSR (D-360 amplifier, 1401 unit; Cambridge Electronic Design, Cambridge, UK). Data were acquired in Spike2 (v10.05).

### 2.5. Analysis

Data analysis was performed in MATLAB (R2021b) and the code is available on GitHub (https://github.com/meaghanspedden/stepping_opm_data (accessed on 29 June 2025)). We used the development version of Statistical Parametric Mapping (SPM; https://github.com/spm (accessed on 30 May 2025)) [33] to perform the source imaging of movement-related beta power.

#### 2.5.1. Preprocessing

The OPM data were imported into SPM and resampled to 1000 Hz. The OPM power spectra were then visually inspected for bad channels (i.e., large deviations from median power and/or manufacturer’s noise floor). For participants 1, 2, and 3, respectively, the final number of channels included was 56, 57, and 162 (i.e., after excluding bad channels).

Harmonic models, i.e., homogenous field correction (HFC) or adaptive multipole models (AMMs), were applied to the OPM data for interference suppression [17,34] depending on the number of OPM channels. HFC was applied to data from participant 1 and 2 (number of channels < 120), whereas AMMs were applied to data from participant 3 (number of channels > 120). For AMMs we also used temporal extension (correlation limit 0.98) [17]. HFC models the interference as a spatially constant field and can substantially reduce environmental noise over a broad range of frequencies [16]. AMMs make not only a model of the external interference, but also the fields arising within the head and a third partition of signal that does not conform to either model; this adds further immunity against magnetic sources of interference [17].

The OPM data were high pass filtered at 5 Hz, then low pass filtered at 45 Hz, and finally, notch filtered at 49–51 Hz to remove residual line noise.

Initially, the OPM and EMG data were epoched into single trials (i.e., each step) based on triggers indicating the appearance of the stepping target. The standing period was defined as −1.9 to −1.4 s before target presentation for all participants. The EMG signals in this interval were visually inspected to confirm that the participants were standing still. The stepping period was defined individually for each participant based on the timing of the first EMG burst, which indicated the swing phase of the step (visual inspection). This period was from 1.5 to 2 s after the go signal for participant 1, from 1.6 to 2.1 s for participant 2, and from 2 to 2.5 s for participant 3. The 500 ms duration was chosen to avoid artifacts related to the heel strike during the stepping period [4].

#### 2.5.2. Source Imaging

We used the DAiSS toolbox for SPM to perform a source analysis of the OPM data during stepping compared with standing, using beamforming [35]. Time windows for standing were −1.9 to −1.4 s before target presentation for all participants. Time windows for stepping differed based on EMG-defined swing phases (see 2.4.1): 1.5–2 s for participant 1, 1.6–2.1 s for participant 2, and 2.0–2.5 s for participant 3 following the go cue.

Beamformer weights were constructed from the pooled covariance for both conditions (stepping and standing; 500 ms windows), i.e., a common filter, for the beta band (15–30 Hz). The source space comprised a 3D grid covering the whole brain volume (bounded by the inner skull) with 10 mm resolution and a single shell volume conductor [36]. Sensor locations and orientations were innately in the MRI space because each scanner cast was constructed from the participant’s MRI. Source orientation was optimized for maximal power [37]. A volumetric beta power image was printed for each trial and condition. Data were transformed into the MNI space in SPM for consistency and ease of interpretation. The transformation was achieved with an affine transformation between fiducials in the native and MNI space. Standing and stepping were compared (within-participant) using a paired *t*-test in SPM with the contrast standing > stepping. Note that stepping data were pooled over different step lengths (preferred step length ± 5 cm. The resulting images were thresholded using the Random Field Theory correction [38] with a significance level of *p* < 0.05 and family-wise error correction.

We also present a spectrogram showing time–frequency responses averaged across participants. To construct this, we extracted the source time series from the coordinate with the maximal t-statistic for the standing vs. stepping contrast in each participant.

#### 2.5.3. Relationships Between Brain Activity and Behavior

To evaluate whether beta band brain activity was related to participant behavior, we performed two exploratory analyses. First, we asked whether the source reconstructed beta band power from the source with maximal t-statistic for the standing vs. stepping contrast in each participant was correlated with the log-transformed step error. We reported correlation coefficients and *p*-values for this analysis.

Second, we examined whole-brain beta band activity by performing a *t*-test for each participant, comparing low-error and high-error trials ([1 −1]) based on a median split. Statistical images were corrected for multiple comparisons using the same approach as for the stepping vs. standing contrast.

## 3. Results

**Figure 3:**
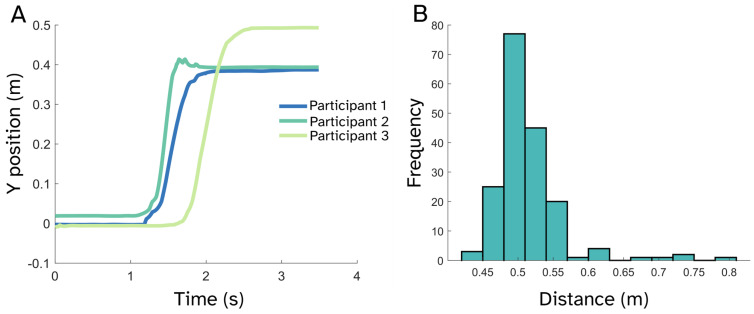
Description of movement during stepping. (**A**) Median trajectory in the anterior–posterior (forward–backward) direction for each participant across steps. (**B**) Distribution of step distances for an exemplar participant (Participant 3). The ‘go’ cue for the step is at t = 1 s.

We investigated whether known physiological signals could be reliably recovered during discrete stepping movements involving large-scale, whole-body translation. The mean anterior–posterior distance traveled per step was 0.5 ± 0.05 m across participants. Figure 3 illustrates the median translation trajectory for each participant as well as the distribution of distances for a single exemplar participant.

**Figure 4:**
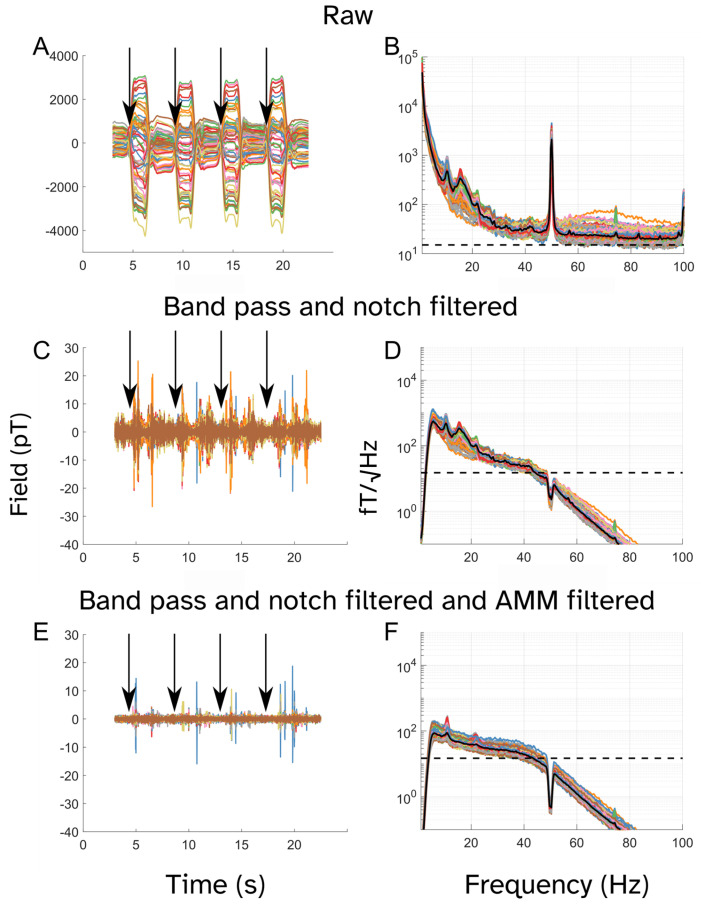
Effect of preprocessing methods on OPM time series and power spectra. The left column shows time series and right column power spectral densities. (**A**,**B**) show raw data. (**C**,**D**) show data after band-pass (5–45 Hz) and notch (49–51 Hz) filtering; (**E**,**F**) show data after band-pass and notch filtering and adaptive multipole modeling (AMM). Data are from participant 3. Arrows (left column) show the movement onset, i.e., four steps are shown. Dotted black horizonal lines show the noise floor and solid black lines show the median values for spectral power (right column) across sensors. Note that the axis scaling in (**A**) is different from (**C**,**E**) as the field magnitude for the raw data was much greater than for the preprocessed data. Similarly, the scale for (**B**) is larger than for (**D**,**F**) to show all data features.

Next, we evaluated the impact of our preprocessing pipelines on the OPM time series. Figure 4 presents the preprocessing pipeline at different stages for a single participant (participant 3).

The Supplementary Material contains additional figures to further illustrate the effects of preprocessing. Supplementary Figure S2 shows the preprocessed channel time-locked to the ‘go’ signal to demonstrate the resulting sensor-level data. Supplementary Figure S3 shows the sensor-level beta band envelope time-locked to the ‘go’ signal for raw and preprocessed data and illustrates that the step-related decrease in beta band power first becomes clear after preprocessing.

We then established the validity of our OPM data by demonstrating movement-related modulations of beta band power while stepping relative to standing. Beta band activity exhibits well-characterized modulations time-locked to the planning, initiation, and execution of movement [30,39] and is a robust effect [28,29] occurring in the sensorimotor cortex.

**Figure 5:**
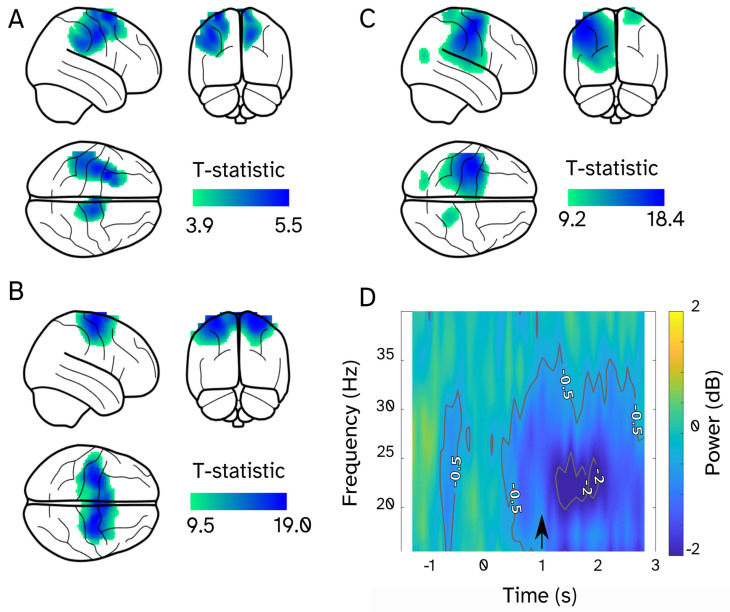
Movement-related beta band modulations. Contrast of stepping vs. standing ([1 −1]) in the beta band (15–30 Hz), quantified as a t-statistic for participants 1, 2, and 3, respectively (**A**–**C**). All significant voxels (FWE < 0.05), which are also above 50% of the maximum t-statistics, are displayed. Stepping data were pooled over different step lengths. Time–frequency spectrum averaged across the three participants for source time series (**D**). The target is presented at t = 0 and the go signal at t = 1.

A significant decrease in beta band power was observed in the sensorimotor cortex across all three participants (Figure 5). The decrease was observed bilaterally for all participants, which is unsurprising given the involvement of both the standing and swinging leg in the step. Maximal t-statistics were localized to MNI: -34, 4, 70 mm for participant 1; 18, −10, 68 for participant 2; and −42, −12, 54 for participant 3.

To assess the impact of preprocessing on the source analysis, we repeated the same procedure using data filtered with only the band-pass and notch filters, excluding AMMs. These findings are summarized in Supplementary Figure S4, which illustrates that while similar source patterns emerge without AMMs, the initial de-noising stage does give rise to a larger peak t-statistic at the source level.

We also considered whether similar results could be achieved with fewer trials. We repeated the analysis using four iterations of a 30-trial block for a single participant. Each iteration revealed consistent suprathreshold activation in the sensorimotor cortex (see Supplementary Figure S5), supporting the robustness of our findings and utility of the paradigm for patient populations.

We then explored possible relationships between brain activity and step error. The correlational analysis suggested the lack of a significant association between step error and beta band activity in the motor cortex, at least for the source where the standing–stepping contrast was maximal (participant 1: r = 0.41, participant 2: r = 0.40, participant 3: r = 0.35; all *p* > 0.05).

The whole-brain contrast between trials with low and high error revealed a significant difference in participant 1, while no activity surpassed the FWE-corrected threshold in participants 2 or 3. The MNI coordinates corresponding to the peak t-statistics are shown in Supplementary Figure S7. For participants 1 and 2, peak activation was located near the central sulcus (MNI: 16, 0, 52 and 50, –4, 40, respectively), whereas for participant 3, it was found in the superior frontal gyrus (MNI: 4, 58, 44).

## 4. Discussion

We found that all three participants exhibited significant decreases in beta band power localized to the sensorimotor cortex during stepping. These results align with the existing literature demonstrating the robust and well-described phenomenon of beta band event-related desynchronization in the sensorimotor cortex during movement compared to rest [40]. The observed bilateral effect is likely attributable to the nature of the whole-body movement, where the stance leg pushes off while the other leg swings forward. These results demonstrate the utility of using OP-MEG as a sensitive tool to study the neural control of human walking. The current study represents a significant advance, demonstrating that it is feasible to obtain meaningful brain-imaging results during whole body movement at the single-participant level using OP-MEG. Single-subject capability is particularly relevant for clinical applications, where cases must often be assessed individually.

In one of the three participants, we also found a significant difference between high and low error steps localized to the sensorimotor cortex. This finding is encouraging, particularly given the limitations of single-subject analyses and the relatively low number of trials per condition due to the median split. This preliminary result suggests that there may be participant-level sensitivity to step precision and warrants further investigation with larger datasets.

Currently, EEG is the most commonly used method for studying brain activity during large-scale movements [41]. The temporal resolution provided by EEG is superior to our current OP-MEG system (which has an intrinsic bandwidth of ~150 Hz (https://quspin.com/products-qzfm/ (accessed on 29 June 2025)). However, because the MEG-forward models depend on fewer conductivity and structural parameters than EEG, MEG models generally provide more robust spatial information [42]. Also, EEG is more susceptible to muscle [7] and head movement artifacts in sensor signals [43]. Consequently, OP-MEG may allow for fundamental improvements in the precision and quality of brain activity recordings attainable during large-scale movement.

Despite these advantages of OP-MEG over EEG, OP-MEG recordings face the challenge of background interference. In order to keep the sensors operating within their range (where the gain is linear), the ambient magnetic field needs to be controlled. In this study, we were able to keep the sensors in range (±4.5 nT) during translations of ~0.5 m through the combination of a high-quality MSR, on-board sensor nulling, and degaussing procedure that strongly decreased the remnant field prior to recordings.

Even in the linear range of sensor operation, interference remains prominent. We addressed this using harmonic models of OPM data [17,34] which used spatial or spatiotemporal models of interference and brain signal to minimize the impact of environmental noise. In practice, these methods have been shown to provide robust, broadband interference suppression [16,44]. The spatial filtering given by the beamformer also provided additional noise immunity. Beamforming theoretically reduces large common artifacts based on the idea that they cannot be explained by local brain sources, an effect which has also been demonstrated experimentally [45].

Taken together, this work supports the utility of OP-MEG in studying the neural control of human walking from both a spatial and temporal perspective. We detected and reproduced known neural markers of movement, providing physiological proof-of-principle supporting the use of this method as a new instrument for studying natural movement dynamics.

Fully realizing the benefits of OP-MEG in naturalistic movement contexts requires a multifaceted approach, combining both technological innovations and methodological frameworks. Movement compensation can be addressed analytically, and current spatial and spatiotemporal filtering approaches have proven effective in mitigating movement-related artifacts and preserving signal fidelity [2,3,4]. However, these techniques depend on maintaining sensor operation within their linear gain range, which can be compromised during large or rapid movements.

This can be addressed through two ambient magnetic field control strategies. Dynamic nulling using dedicated coil systems [5,6] enables real-time suppression of environmental magnetic interference and helps maintain sensor linearity by canceling the ambient field. Closed-loop operation approaches [7] further improve this capability by continuously adapting to changes in the ambient field, expanding the effective dynamic range of the system.

Together, these technological and methodological advances form a complementary framework that positions OP-MEG as a powerful tool for capturing high-fidelity brain activity in naturalistic and dynamic environments, providing new possibilities for cognitive and clinical neuroscience research.

The prospect of recording MEG during human walking has exciting implications for basic research and clinical applications in the field of sensorimotor control. MEG is particularly well-suited to characterize spatiotemporal dynamics of brain networks, which is key because how brain regions and their neuronal populations interact to govern movement remains an open question [46]. For example, this method may help us to understand key questions such as how gait is adapted to environmental demands, which mechanisms mediate the recovery of walking function after neurological injury such as stroke or Cerebral Palsy, and gait problems in neurodegenerative disorders such as Parkinson’s disease. Further, this technique may allow exploration of the neural basis of individual variability in walking patterns and ability. A particularly useful application may be to understand how spinal cord networks interact with cerebral control to coordinate walking. Recent work showing concurrent recordings of the brain and spinal cord activity using OPMs [47] suggests that this method has the potential to begin addressing such fundamental questions in human gait research and lead to transformative insights into the physiological and pathological mechanisms of walking.

## Data Availability

The data can be found on Mendeley Data with doi 10.17632/p3dfxmky46.4. The MATLAB code for our validation analysis is available on GitHub (https://github.com/meaghanspedden/stepping_opm_data (accessed on 29 June 2025)).

## Supplementary Materials

The following supporting information can be downloaded at: https://www.mdpi.com/article/10.3390/s25134160/s1, Figure S1: raw time domain (A) and frequency domain (B) empty room data acquired prior to recording from participant 1. Figure S2: Single-trial time-domain data time-locked to target presentation. Figure S3:Beta envelope for a single sensor for participant 3 over the left sensorimotor region for raw and processed data. Figure S4: Source reconstruction for participant 3 following band-pass and notch filtering and following the full preprocessing pipeline. Figure S5: Suprathreshold t-statistics (FWE-corrected) for four iterations of 1 block. Figure S6: The influence of different thresholding techniques and bi- vs. triaxial arrays. Figure S7: Locations of peak t-statistics for the contrast of steps with low- and high-error trials for each participant.

## Abbreviations

The following abbreviations are used in this manuscript:

OPM	Optically pumped magnetometer
MEG	Magnetoencephalography
MSR	Magnetically shielded room
