# Supplementary material for: Using Wearable MEG to Study the Neural Control of Human Stepping
Source: Sensors (Basel). 2025 Jul 4;25(13):4160. doi: 10.3390/s25134160 (PMC12252452; doi:10.3390/s25134160)
Supplement: Supplementary file 1 [file sensors-25-04160-s001.zip › sensors-3662451-supplementary.pdf]

Supplementary material: **Using wearable MEG to study the neural control of human stepping**

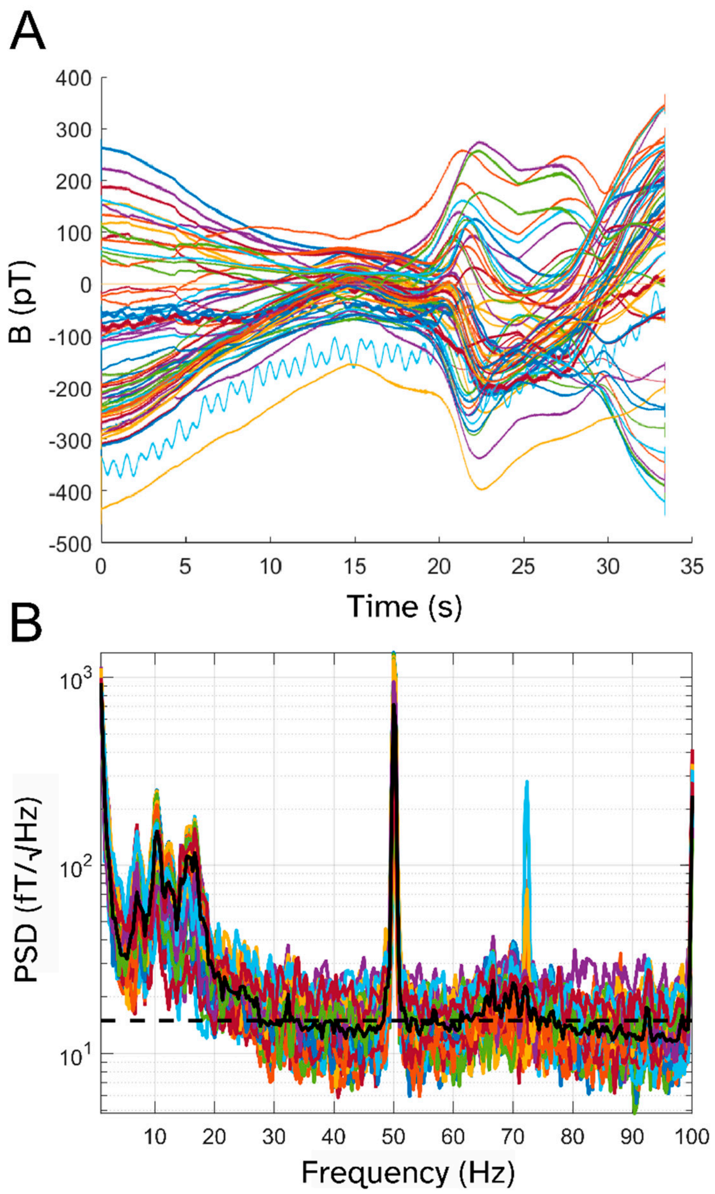

Supplementary Figure 1: raw time domain (A) and frequency domain (B) empty room data acquired prior to recording from participant 1. The dotted line shows the 15fT/sqrt(Hz) white noise level specified by the manufacturer.

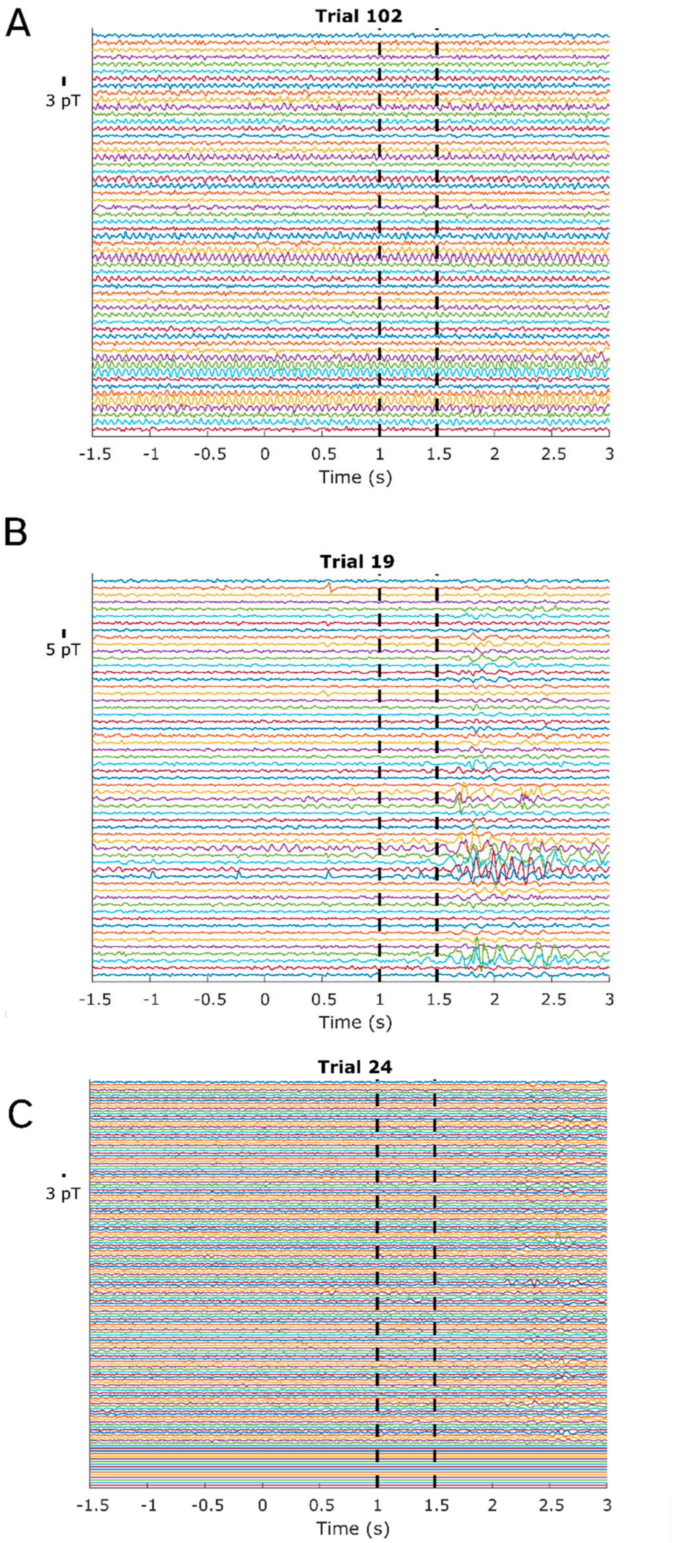

Supplementary Figure 2: Single-trial time-domain data time-locked to target presentation. Vertical lines indicate the (onset and offset) analysis periods for stepping roughly corresponding to the swing phase. A–C show sensor-level data for participants 1–3, respectively. For each participant, the displayed trial was selected as the one whose beta-band envelope was most strongly correlated with the average envelope.

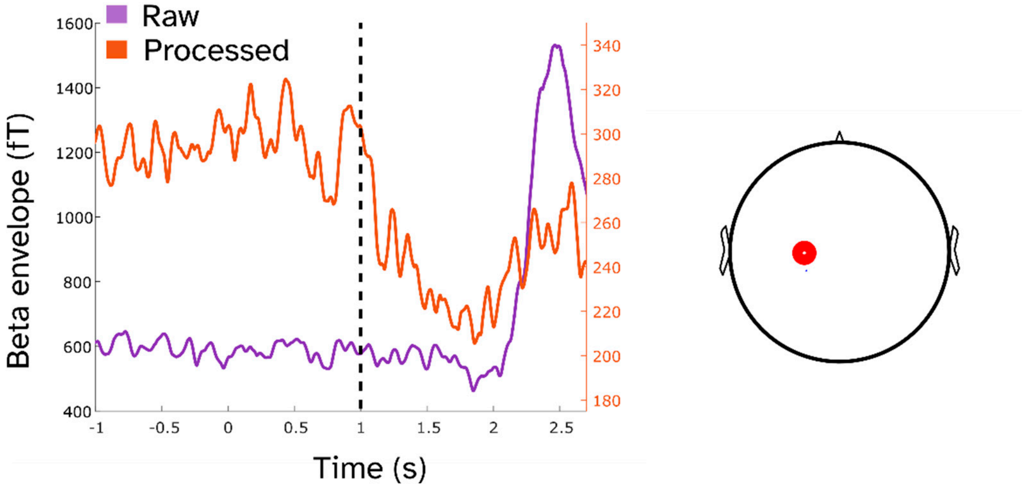

Supplementary Figure 3. Beta envelope for a single sensor for participant 3 over the left sensorimotor region for raw and processed data. The position of this sensor is visualised on the topoplot on the right. Note that the axes are scaled differently so all data features are visible. The 'go' signal appears at t=1, hence the modulation in the raw data is probably due to a step being taken.

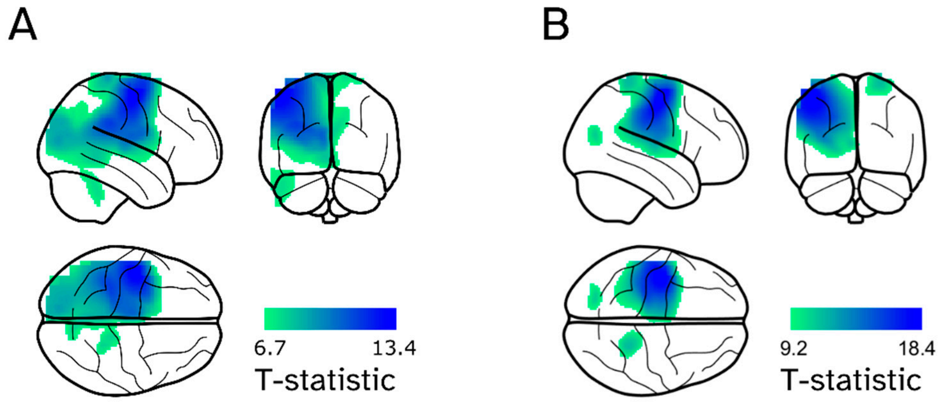

Supplementary Figure 4. Source reconstruction for participant 3 following band-pass and notch filtering (A) and following the full preprocessing pipeline (band-pass and notch filtering and AMM (B). Suprathreshold (FWE-corrected) t-statistics are shown.

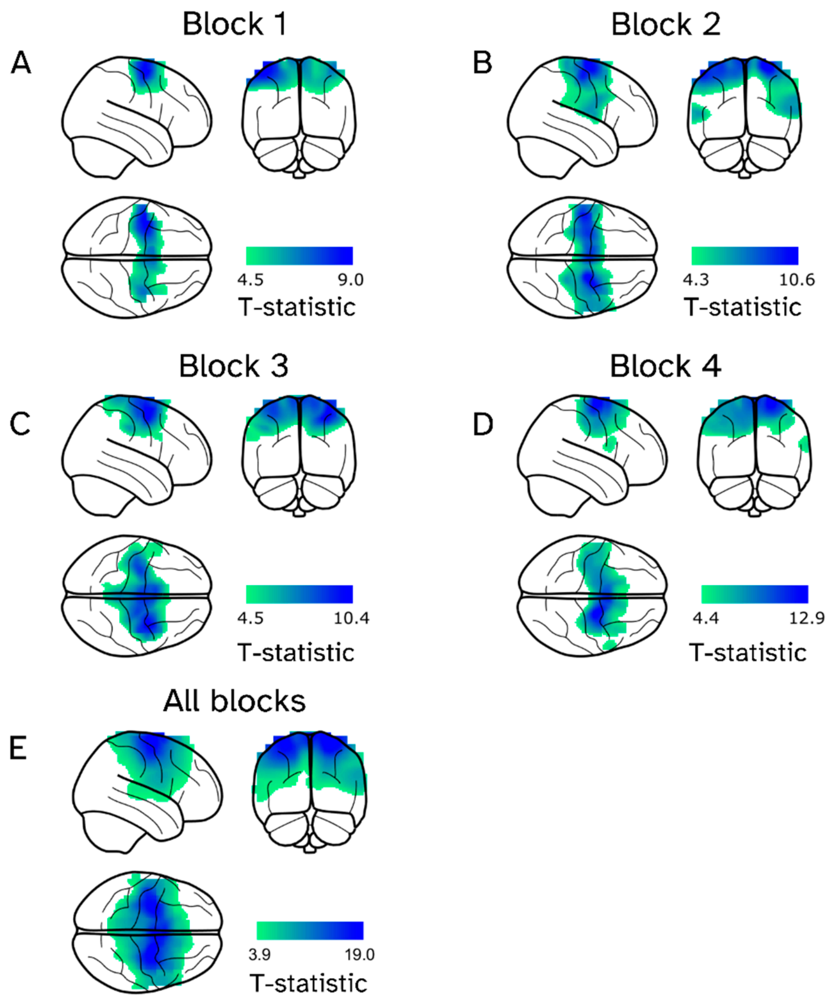

Supplementary Figure 5. Suprathreshold t-statistics (FWE-corrected) for four iterations of 1 block (30 trials; participant 2) (A-D). This suggests that similar results can be achieved with fewer trials. The results for all blocks (5; 150 trials) are shown in (E). Note that the maximal t-statistic is considerably greater for (E).

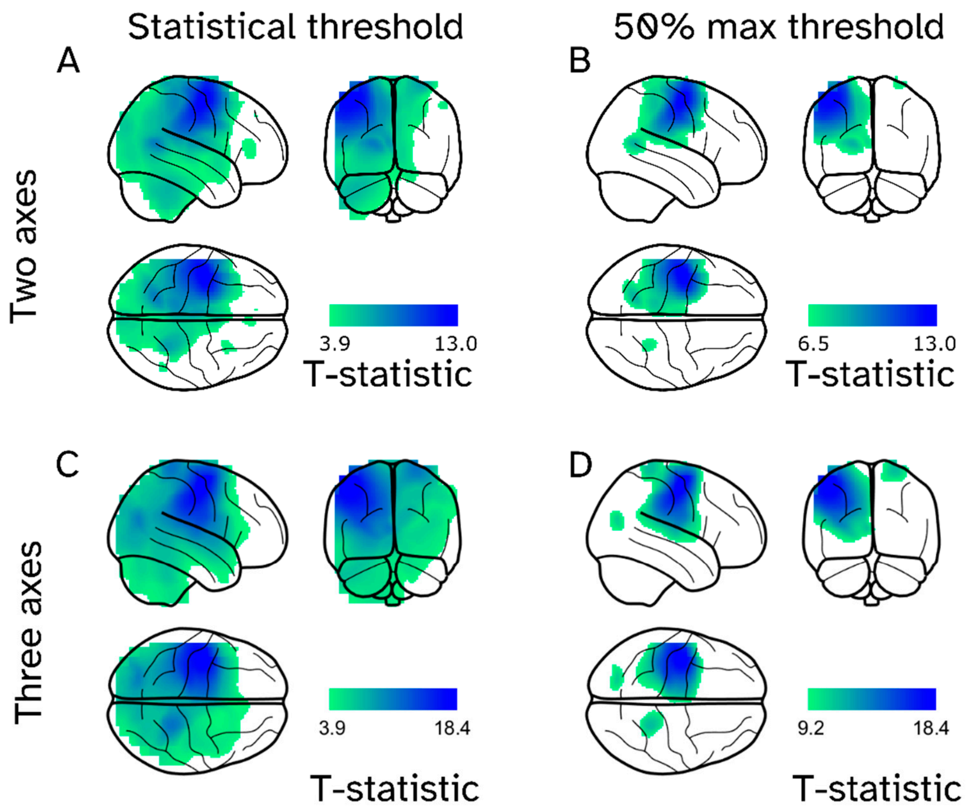

Supplementary Figure 6. The left and right columns (respectively) show the influence of different thresholding techniques ($p<0.05$, FWE corrected) or 50% of maximum t-statistic. The top and lower rows show the influence of using a bi (top) or triaxial (lower) channel array. In the bi-axial case one of the tangentially oriented channels has been removed. Data is from participant 3.

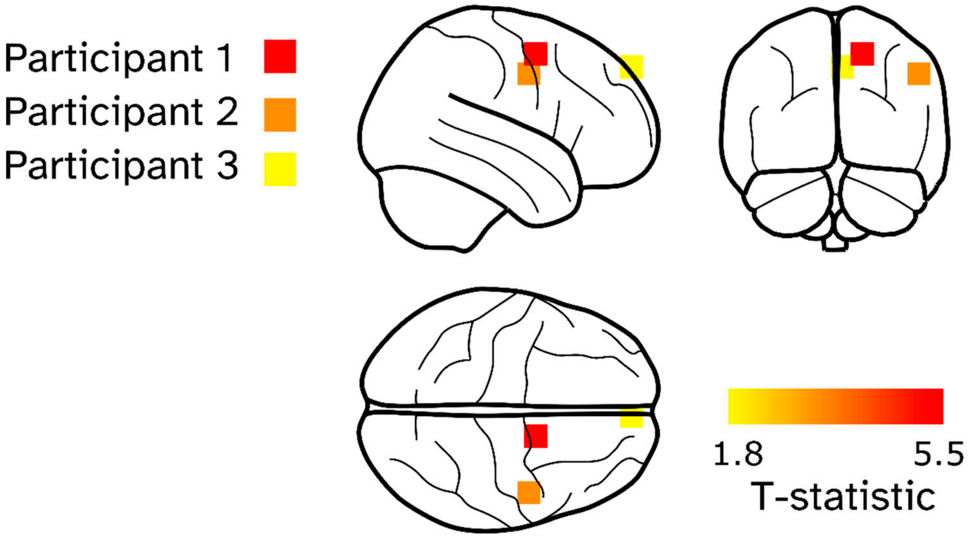

Supplementary Figure 7. Locations of peak t-statistics for the contrast of steps with low- and high-error trials for each participant. Note that only participant 1 exhibited a significant difference between the two conditions after FWE correction, while t-statistics for participants 2 and 3 did not exceed the corrected threshold.
